# Influence of Dietary Diversity and Its Associated Factors Among Human Immunodeficiency Virus–Infected Adult Patients Receiving Antiretroviral Therapy in Northwest Ethiopia

**DOI:** 10.1155/arat/9916549

**Published:** 2025-08-04

**Authors:** Yihenew Sewale, Kassahun Dires Ayenew

**Affiliations:** ^1^Department of Adult Health Nursing, Asrat Woldeyes Health Science, Debre Birhan University, Debre Berhan, Ethiopia; ^2^Department of Pharmacy, Asrat Woldeyes Health Science Campus, Debre Berhan University, Debre Birhan, Ethiopia

**Keywords:** acquired immunodeficiency syndrome, antiretroviral therapy, dietary diversity, Ethiopia, human immunodeficiency virus

## Abstract

**Introduction:** Influence of dietary diversity is particularly concerning for individuals with human immunodeficiency virus (HIV), as they are more vulnerable to opportunistic infections. However, information on influence of dietary diversity in this study area remains scarce. Thus, this study aims to assess the influence of dietary diversity and its associated factors among HIV-infected adults receiving antiretroviral therapy (ART) in Ethiopia.

**Methods:** An institution-based cross-sectional study was conducted among 412 participants between February and March 2023. Data were collected using an interviewer-administered structured questionnaire and a standardized checklist for ART. Data entry was performed using EpiData Version 3.1, and analysis was conducted using STATA Version 25. Multivariable logistic regression was used to identify factors associated with dietary diversity, with adjusted odds ratios (AORs) reported at a 95% confidence interval (CI) and a significance level of *p* < 0.05.

**Results:** The proportion of patients with adequate dietary diversity was 218 (52.9%; 95% CI: 48.1–58). Factors significantly associated with dietary diversity included educational status (AOR: 0.414, 95% CI: 0.174–0.985), family size of 4–6 (AOR: 1.87, 95% CI: 1.18–2.95), and WHO clinical stage III or IV (AOR: 1.19, 95% CI: 1.09–2.34).

**Conclusions:** The study found that nearly half of HIV-infected adult patients had an undiversified diet. Occupation being housewives and drivers, educational status of unable to write and read, WHO advanced HIV stage III and IV, and family size of 4–6 were statically significant factors associated with undiversified diet. We strongly recommend that policymakers, researchers, and nongovernmental organizations collaborate to implement holistic nutritional interventions to address dietary challenges and improve the overall health of people living with HIV/AIDS.

## 1. Introduction

Globally, an estimated 37.9 million people are living with human immunodeficiency virus (HIV), with over 23.3 million receiving antiretroviral therapy (ART) [1]. Dietary diversity and HIV/AIDS are interrelated, forming a vicious cycle in which both conditions exacerbate one another by weakening the immune system [2]. Undernutrition and HIV share similar cellular effects, including a decline in CD4 T-cells, suppression of delayed hypersensitivity, and abnormal B-cell responses [3]. These issues arise due to inadequate dietary intake and altered metabolic conditions, leading to an imbalance of energy and nutrients, even in patients undergoing ART [4].

Dietary diversity refers to the number of different foods and food groups consumed by an individual over 24 h, including foods eaten outside the home. It is commonly categorized as an undiversified diet (≤ 5 food groups) or a diversified diet (≥ 6 food groups) [5]. At the individual level, dietary diversity score serves as a proxy indicator of adequate energy and micronutrient intake [6]. Consuming a variety of food groups is an internationally accepted recommendation for a healthy diet and positive health outcomes [7]. The consequences of an undiversified diet are particularly critical for HIV-infected individuals, as they are more susceptible to opportunistic infections (OIs). Proper dietary management is essential for maintaining their ability to carry out daily activities and improving their overall well-being [8].

Nutrition is a key component of comprehensive ART care, especially in low- and middle-income countries where food insecurity and dietary deficiencies are prevalent [3]. Access to a diversified diet is crucial for meeting basic health, growth, and development needs, yet this remains a longstanding challenge in many African nations [8].

In addition to ART, comprehensive care includes Hyperactive Antiretroviral Therapy (HAART), nutritional assessment, counseling, and ongoing patient monitoring [9].

The World Health Organization (WHO) recommends increased energy intake for HIV-infected individuals receiving ART, depending on their symptomatic stage. Asymptomatic patients require 10% more energy intake than noninfected individuals, while symptomatic patients need 20%–30% more. In cases of severe malnutrition, energy requirements can increase by 50%–100%, highlighting the impact of an undiversified diet on disease progression [10]. Studies conducted in Nigeria and Uganda found that the prevalence of undiversified diets among HIV-infected individuals receiving ART was 58.8% and 62.3%, respectively [11, 12].

In Ethiopia, limited research has examined dietary diversity among HIV-infected adults receiving ART, with reported rates ranging from 28.7% to 70.5% [13–16]. Contributing factors for an undiversified diet include mobile phone ownership, media exposure, and access to nutritional counseling [16], duration of ART use [14], occupation, educational status [15], sex, marital status [17], and employment status [13].

The Ethiopian government has implemented consolidated national guidelines for ART care and treatment, incorporating nutritional support to enhance the quality of care for people living with HIV. However, dietary diversity remains a significant issue, with 67.6% of HIV-infected patients on ART reportedly consuming an undiversified diet [15]. Additionally, there is limited information on the effects of dietary diversity among HIV-infected patients receiving ART in Ethiopia [14, 16, 17]. This study aims to assess individual dietary diversity (IDD) and its associated factors among HIV-infected adults attending the ART clinic in Northwest Ethiopia.

## 2. Materials and Methods

### 2.1. Study Design, Period, and Setting

An institution-based cross-sectional study design was employed and conducted over a 2-month period, from February to March 2023. The study population consisted of adult HIV/AIDS patients who were receiving ART and follow-up care at a single comprehensive specialized hospital located in Northwest Ethiopia. This hospital is one of the major health institutions in the region that provides ART services and caters to a large number of HIV-infected individuals.

On average, the ART clinic of the hospital serves approximately 3220 HIV-infected patients per month. Of these, around 1220 are male and 2000 are female adult patients. All individuals included in this study were HIV-positive adults who were on ART and attending routine follow-up visits during the data collection period. Participants were recruited systematically from this clinic to ensure representativeness and to meet the study's inclusion criteria [18].

### 2.2. Population

The source and study population included all HIV-infected adults receiving ART and available during the data collection period at the ART clinic facility.

### 2.3. Sample Size Determination and Sampling Procedures

The sample size was determined using a single population proportion formula, considering factors of dietary diversity among adult HIV-infected patients receiving ART. The calculated sample size was 412 patients [14]. Patients were selected using a systematic sampling technique. The sampling interval (*k*) was determined based on the total number of ART clinic patients per month (*N* = 3220) and the required sample size (*n* = 412), calculated as *k*=*N*/*n*=*k*=(3220/412) ≈ 8, and participants were selected from daily follow-up visits using this interval.

### 2.4. Variables of the Study

#### 2.4.1. Outcome Variable

• Dietary diversity (diversified vs. undiversified).

#### 2.4.2. Independent Variables

The exposure variables are sociodemographic factors of age, sex, residence, occupation, educational status, and family size, clinical-related factors of CD4 count, viral load, ART adherence, WHO clinical stage, OIs, ART duration, ART regimen, prophylactic treatments, and comorbidities, and lifestyle-related factors including smoking, alcohol consumption, physical inactivity, and khat chewing.

### 2.5. Operational Definitions

Dietary diversity refers to a number of different foods and food groups consumed by an individual over a 24-hour period, including foods consumed outside the home. Dietary diversity is classified as follows, low dietary diversity (IDD): 0–4 food groups, moderate dietary diversity (IDD): 5–9 food groups, and high dietary diversity (IDD): 10–14 food groups or undiversified diet: ≤ 5 food groups and diversified diet: ≥ 6 food groups [5].

Adherence to ART is assessed based on the recent adherence status recorded via pill count of good adherence: ≥ 95% of doses taken, fair adherence: 85%–94% of doses taken, and poor adherence: < 85% of doses taken [2].

### 2.6. Data Collection Procedures and Quality Control

Data were collected using an interviewer-administered structured questionnaire. The questionnaire was developed based on previous literature [5, 14, 19], a standardized ART checklist, and standardized verbal questionnaires from the Food and Agriculture Organization (FAO) [20]. Data collection involved face-to-face interviews with all patients and a review of their medical charts. Three bachelor's degree–holding nurses, currently working at the ART clinic of Debre Markos Comprehensive Specialized Hospital, were recruited as data collectors.

### 2.7. Quality Control Measures

The questionnaire was initially developed in English, then translated into Amharic, and back-translated into English by expert of language to ensure accuracy, and the tool was pretested on 10% of the total sample at Debre Markos Health Center to assess clarity and reliability. Daily supervision also was conducted by the principal investigator to ensure adherence to data collection protocols, and a two-day training session was provided for both data collectors and the supervisor, covering the study objectives, significance, and study variables. The supervisor and principal investigator carefully monitored the completeness and consistency of the data throughout the collection process.

### 2.8. Data Analysis and Interpretation

Data entry was performed using EpiData Version 3.1, and statistical analysis was conducted using STATA Version 14.1. Descriptive statistics were used to summarize categorical variables, and results were presented using tables and graphs and continuous variables were described using measures of central tendency (mean) and measures of dispersion (standard deviation).

## 3. Results

### 3.1. Sociodemographic Characteristics of the Study Participants

A 100% response rate was achieved in this study, indicating full participation of all selected respondents. Among the participants, 56.3% were female, reflecting a slightly higher representation of women in the study population. Furthermore, a significant majority of the participants (82.2%) were residents of urban areas, suggesting that the study population was predominantly urban-based. The mean age of the study participants was 43.12 years, with a standard deviation of ±11.29 years. In terms of marital status, approximately 43.9% of the participants were married, suggesting that nearly half of the respondents lived with a partner or spouse.

Regarding employment status, 21.8% of the respondents were government employees, while the rest were self-employed, unemployed, or engaged in informal sectors. Educational status revealed that 30.6% of the participants were unable to read and write, indicating a notable proportion of the population with low literacy levels.

Family size distribution showed that 61.9% of the respondents had a family size of 1 to 3 members, reflecting relatively small household sizes. In terms of nutritional support and counseling, a majority of the participants—69.9%—reported that they were not receiving any form of nutritional support or dietary counseling, highlighting a critical gap in the integration of nutritional care within HIV treatment services (Table 1).

### 3.2. Clinical Characteristics of the Study Participants

Out of the total study participants, the majority of 324 patients (83%) were classified as being in the early stage of HIV disease, based on the WHO clinical staging criteria. However, more than one-fourth (27.9%) of the participants had a CD4 cell count of less than 500 cells/μL, indicating varying degrees of immune suppression among the study population.

In terms of treatment duration, 38.4% of the respondents had been receiving ART for 10 years or more, reflecting long-term engagement in HIV care. Additionally, a significant majority—80.6%—were receiving cotrimoxazole preventive therapy (CPT), which is recommended to prevent OIs among people living with HIV. Regarding comorbid conditions, 5.1% of the patients were diagnosed with diabetes mellitus, of which a striking 85.3% had type II diabetes mellitus, indicating that noncommunicable diseases are present, though relatively uncommon, in this population.

Adherence to ART was found to be suboptimal in a portion of the participants: 11.9% of patients were classified as having fair or poor adherence to their ART regimen. Notably, 97.8% of the study participants had not developed any comorbid conditions, suggesting that a large majority were not affected by other chronic or infectious diseases in addition to HIV. Regarding the ART regimen, 32.5% of the patients were taking the 1e regimen, which includes tenofovir disoproxil fumarate (TDF), lamivudine (3TC), and efavirenz (EFV)—a widely used fixed-dose combination in HIV management (Table 2).

### 3.3. Dietary Diversity of HIV-Infected Patients

Approximately one-third of the patients, 142 (34.5%), had a low IDD, consuming only 0–4 food groups in the 24 h prior to data collection. More than half of the patients, 249 (60.4%), had a moderate IDD, consuming 5–9 food groups. Only a small proportion, 21 patients (5%), exhibited a high IDD, with intake from 10 to 14 food groups within the same period. In terms of specific food groups consumed, the majority of patients reported eating cereals (81.6%) and fats and oils (81.1%). More than half of the participants also consumed white tubers (60%) and other vegetables (74%).

**Figure 1:**
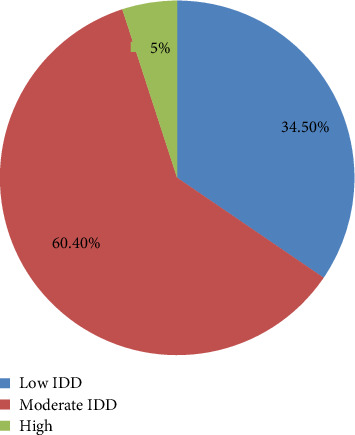
Individual dietary diversity characteristics of the HIV-infected adult patients receiving ART, Northwest Ethiopia, 2023.

A majority of the patients did not consume food groups such as vitamin A–rich fruits (74%), other fruits (86.2%), iron-rich organ meats (81.1%), flesh meats (78.2%), eggs (87.1%), fish (94.4%), and milk and milk products (85.2%). These findings indicate a diet heavily reliant on staple and energy-dense foods, with limited intake of protein-rich and micronutrient-dense food groups, which could contribute to poor nutritional status and related health outcomes among the patients (Figure 1).

### 3.4. Associated Factors With Undiversified Diet Among HIV Infected Patients

Thirteen variables (*p* value < 0.2) were selected for multivariable logistic regression analysis. In the multivariable analysis, only four variables were identified as statistically significant factors associated with an undiversified diet.

Accordingly, patients who were housewives and drivers were 59% less likely to eat a diversified diet than governmental workers (adjusted odds ratio [AOR]: 0.41, 95% confidence interval [CI]: 0.17–0.98). Similarly, patients who were unable to read and write were 86% less likely to eat a diversified diet compared to those with a college or university education (AOR: 0.14, 95% CI: 0.05–0.37).

Patients with a family size of 4–6 were 1.87 times more likely (AOR: 1.87, 95% CI: 1.18–2.95) to consume an undiversified diet than those with a family size of fewer than three. Additionally, patients with a WHO stage of III or IV were 1.19 times more likely (AOR: 1.19, 95% CI: 1.09–2.34) to have an undiversified diet than those with a WHO stage of I or II (Table 3).

## 4. Discussion

The magnitude of undiversified diet among HIV infected patients was 52.9% (95% CI: 48.1–58). This finding aligns with previous studies conducted in Metema (58.8%) [13] and Nigeria (62.3%) [11]. However, it is lower than results reported in Hosanna town (67.9%) [21], Debre Tabor Hospital (67.6%) [15], Motta town (70.5%) [16], and Ambo town (71%) [17].

Conversely, it is higher than findings from Dembiya,Ethiopia, (11.3%) [22], Uganda (14.7%) [23], and Hiwot Fana and Dilchora Hospitals (28.7%) [14]. These variations may be due to differences in sample size, study settings, dietary diversity cutoff points, seasonal variations, ART duration, ART regimens, and disease stage. Additionally, participant characteristics may also contribute to these discrepancies.

Factors associated with undiversified diet were education level (unable to write and read), occupation (housewives and drivers), family size (4–6), and HIV stage of advanced WHO stage of HIV found to be statistically significant in influencing dietary diversity.

Being a housewife or driver was also significantly associated with undiversified diets, consistent with studies conducted in Metema [13], Jimma [24], Debre Tabor Hospital [15], and India. [25]. This could be due to income stability—individuals with regular income sources are more likely to purchase adequate and diverse foods, and food security among these groups may increase their capacity to consume a diverse diet at both individual and household levels [26, 27].

Being unable to read and write was significantly associated with undiversified diets. This finding is consistent with studies conducted in Jimma [24], Metema [13], Debre Tabor [15], and Amatole and Nyandeni districts, South Africa [28]. Possible explanations include limited access to income-generating opportunities and poor understanding of nutritional education and counseling, and less-educated individuals may also lack knowledge about dietary practices and meal planning based on nutritional requirements [29].

Participants with larger family sizes (4–6 members) were more likely to consume a diversified diet compared to those with smaller family sizes (1–3 members). A possible explanation is that larger families require greater food variety, leading to higher dietary diversity.

An advanced WHO HIV stage was significantly associated with undiversified diets, similar to findings in Hosanna town [21] and Zimbabwe. [30]. Asymptomatic HIV-infected patients on ART need to increase their energy intake by 10% compared to noninfected individuals. Symptomatic patients or those with OIs require a 20%–30% increase in energy intake, while those with severe malnutrition may need a 50%–100% increase [10]. This dietary challenge may be due to loss of appetite, mouth ulcers, food insecurity, malabsorption of macronutrients, and altered metabolism.

### 4.1. Strengths and Limitations of the Study

This is mainly related to the nature of the cross-sectional design, which does not demonstrate a cause–effect relationship. There is also recall bias.

## 5. Conclusions

Therefore, nutrional counseling, education, close flow up of patients, and intervening of the existing problems are important for addressing the problems. We also strongly recommend that political leaders, researchers, and nongovernmental organizations come together to integrate holistic nutritional interventions to address nutritional problems.

## Figures and Tables

**Table 1:** Sociodemographic characteristics of HIV-infected adult patients receiving ART in Northwest Ethiopia, 2023.

Variables	Number (*N*)	Percent (%)
*Age groups*		
18–34 years	86	20.9
35–45 years	164	39.8
> 45 years	162	39.3

*Marital status*		
Married	181	43.9
Single	45	10.9
Divorced	97	23.5
Windowed	89	21.6

*Occupation*		
Daily laborer	65	15.8
Governmental employee	90	21.8
Private worker	81	19.7
Farmer	44	10.7
Merchants	69	16.7
Housewives and drivers	63	15.3

*Education*		
Unable to write and read	126	30.6
Able to read and write	36	8.7
Primary school	94	22.8
Secondary school	102	24.8
College/university	54	13.1

**Table 2:** Clinical-related characteristics of the HIV-infected adult patients receiving ART in Northwest Ethiopia, 2023.

Variables	Number (*N*)	Percent (%)
*HIV stage*		
Early (I & II)	342	83
Advanced III & IV	70	17

*CD4 (cell/mm* ^3^)		
< 500	115	27.9
≥ 500	297	72.1

*Viral load in the blood*		
< 1000	372	90.3
≥ 1000	40	9.7

*Comorbidities*		
Yes	9	2.2
No	403	97.8

*Opportunistic infection*		
Yes	126	30.6
No	286	69.4

*Hyperactive antiretroviral therapy regimen*		
1e (TDF + 3TC + EFV)	134	32.5
IJ (TDF + 3TC + DTG)	157	38.1
2h (TDF + 3TC + ATV/r) & 2f (AZT + 3TC + ATV/r) & 2e (AZT + 3TC + LPV/r) & ABC + 3TC + ATV/r	74	18
1c (AZT-3TC-NVP) & 1f (TDF + 3TC + NVP)	29	7
Others	18	4.4

*Drugs for comorbidities*		
Yes	30	7.3
No	382	92.7

**Table 3:** Multivariable logistic regression analysis of associated factors of dietary diversity among HIV-infected adult patients receiving ART in Northwest Ethiopia, 2023.

Variables	Dietary diversity		COR (95%)	AOR (95%)
	Undiversified	Diversified		
*Educational status*				
Unable to write and read	87	39	1	1
Able to read and write	21	15	0.35 (0.14, 0.85)	0.33 (0.11, 0.98)
Primary school	48	46	0.47 (0.23, 0.96)	0.31 (0.12, 0.75)
Secondary school	44	58	0.65 (0.33, 1.31)	0.39 (0.17, 0.92)
College/university	18	36	0.22 (0.11, 0.44)	0.14 (0.05, 0.37)

*Occupation*				
Daily laborer	40	25	0.51 (0.24, 1.08)	0.75 (0.33, 1.69)
Governmental worker	43	47	0.49 (0.45, 0.78)	0.41 (0.17, 0.98)
Private worker	44	37	0.69 (0.34, 1.39)	0.70 (0.32, 1, 54)
Farmer	34	10	0.24 (0.09, 0.59)	0.31 (0.11, 1.89)
Merchants	30	39	1.06 (0.51, 2.21)	0.83 (0.37, 1.88)
Housewives and drivers	27	36	1	1

*Family size*				
1–3	146	109	1	1
4–6	65	79	1.62 (1.07, 2.45)	1.87 (1.18, 2.95)
> 6	7	6	1.14 (0.37, 3.51)	1.37 (0.39, 4.74)

*WHO stage*				
Early (I & II)	188	154	1	1
Advanced III & IV	30	40	1.62 (1.06, 2.73)	1.19 (1.09, 2.34)

## Data Availability

Data are available on request from the authors.

## Nomenclature

AIDS: Acquired immunodeficiency syndrome
ART: Antiretroviral therapy
IDD: Individual dietary diversity
PLHIV: People living with human immunodeficiency virus
WHO: World Health Organization
